# Supplementary material for: Elongator mutation in mice induces neurodegeneration and ataxia-like behavior
Source: Nat Commun. 2018 Aug 10;9:3195. doi: 10.1038/s41467-018-05765-6 (PMC6086839; doi:10.1038/s41467-018-05765-6)
Supplement: Supplementary file 1 — Supplementary Information [file 41467_2018_5765_MOESM1_ESM.pdf]

Supplementary information

Elongator mutation in mice induces neurodegeneration and ataxia-like behavior

Kojic *et al*.

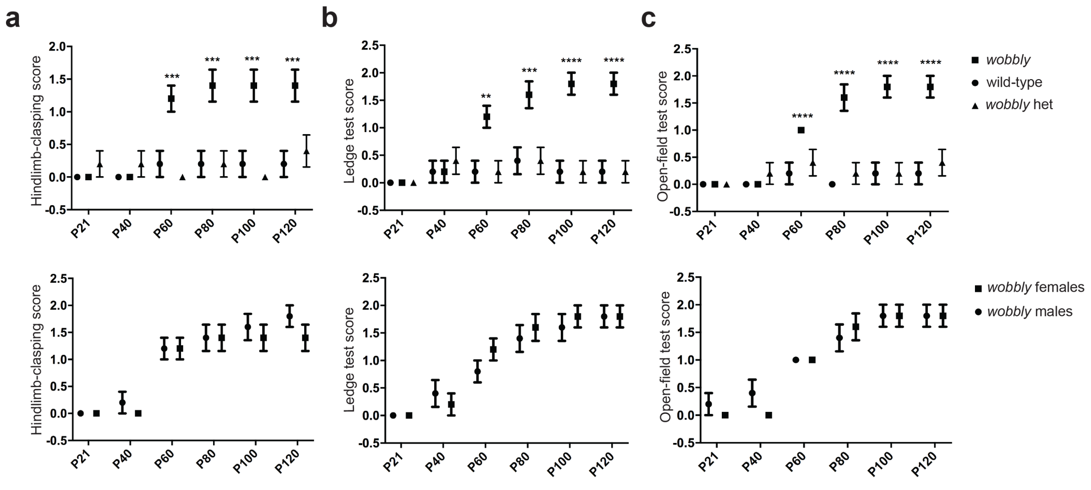

Supplementary Figure 1. Behavioral testing of *wobbly* mice according to the simple composite phenotype scoring system for cerebellar ataxia. (**a**) Hindlimb-clasping, (**b**) ledge and (**c**) open-field test analysis of the *wobbly* phenotype (homozygous *wobbly* animals are presented as *wobbly* and heterozygous as *wobbly* het) is presented relative to wild-type. For upper panels $n = 10$ (5 males and 5 females) for each of the genotypes. For lower panels $n = 5$ for each of the sexes. Statistical evaluation: two-way ANOVA and Sidak's multiple comparisons test. Statistically significant differences are indicated (** $P \leq 0.01$; *** $P \leq 0.001$; **** $P \leq 0.0001$). Data represent mean ± SEM.

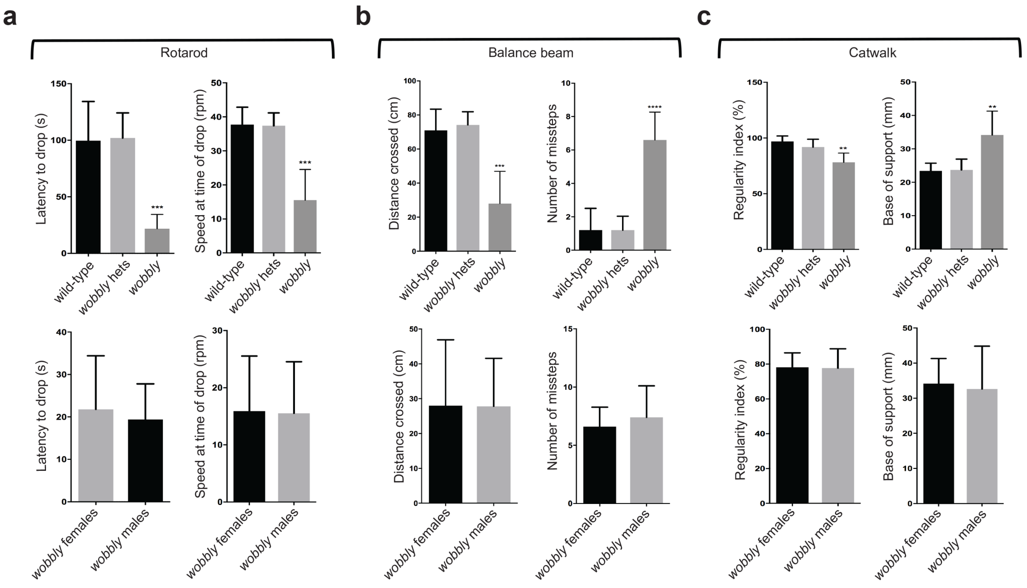

Supplementary Figure 2. Behavioral testing of P100 *wobbly* mice using rotarod, balance beam and Catwalk system. (**a**) Analysis of the *wobbly* phenotype (homozygous *wobbly* animals are presented as *wobbly* and heterozygous as *wobbly* het) relative to wild-type controls using rotarod, (**b**) balance beam and (**c**) Catwalk system is presented. For upper panels n = 10 (5 males and 5 females) for each of the genotypes. For lower panels n = 5 for each of the sexes. Statistical evaluation: **upper panel**, two-way ANOVA and Sidak's multiple comparisons test; **lower panel**, two-tailed t-test. Statistically significant differences are indicated (** $P \leq 0.01$; *** $P \leq 0.001$; **** $P \leq 0.0001$). Data represent mean ± SEM.

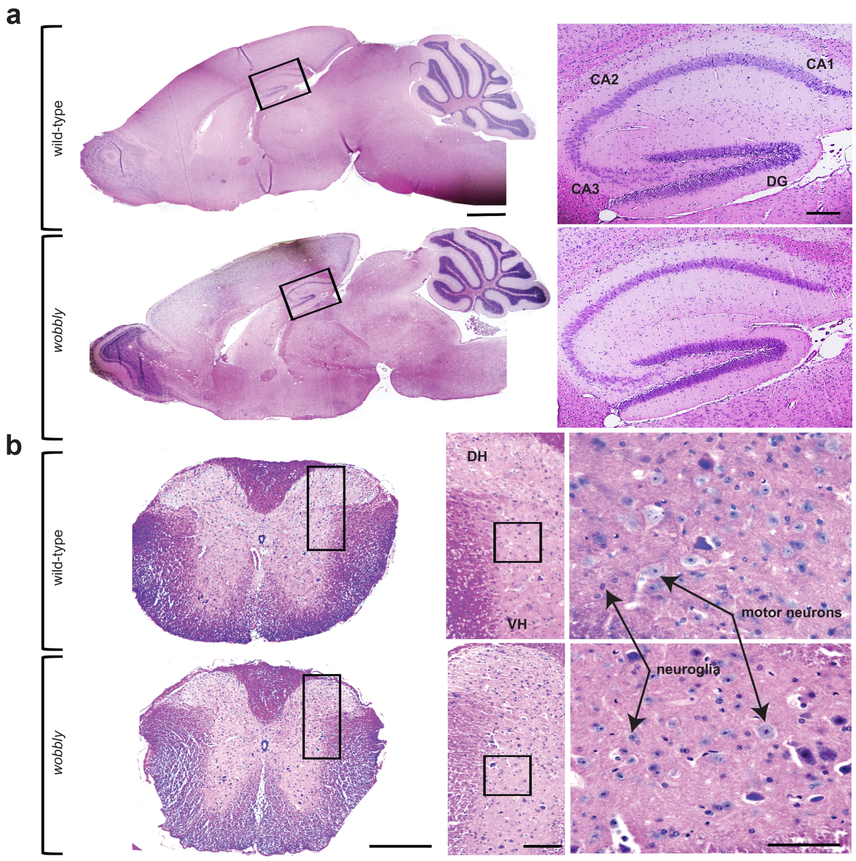

Supplementary Figure 3. Gross morphology of the brain and spinal cord in P120 *wobbly* mice. (**a**) H&E staining of whole brain sagittal sections of *wobbly* and control animals are presented in the left and hippocampus in the right panel. (**b**) H&E staining of transverse spinal cord sections of mutant and wild-type mice. Black rectangles represent magnified areas. n = 5 for each of the genotypes; representative images are shown. Abbreviations: CA – *Cornu Ammonis*; DG – dentate gyrus; DH – dorsal horn; VH – ventral horn. Other analyzed tissues include: testis, preputial gland, prostate, uterus, ovaries, vagina, urinary bladder, liver, gall bladder, cecum, colon, spleen, pancreas, mesenteric lymph node, stomach, duodenum, jejunum, ileum, kidney, adrenal, salivary glands, lymph nodes, thymus, lungs, heart, skin, eyes, brain, spinal cord, skeletal muscle and skeletal tissue (data not shown).

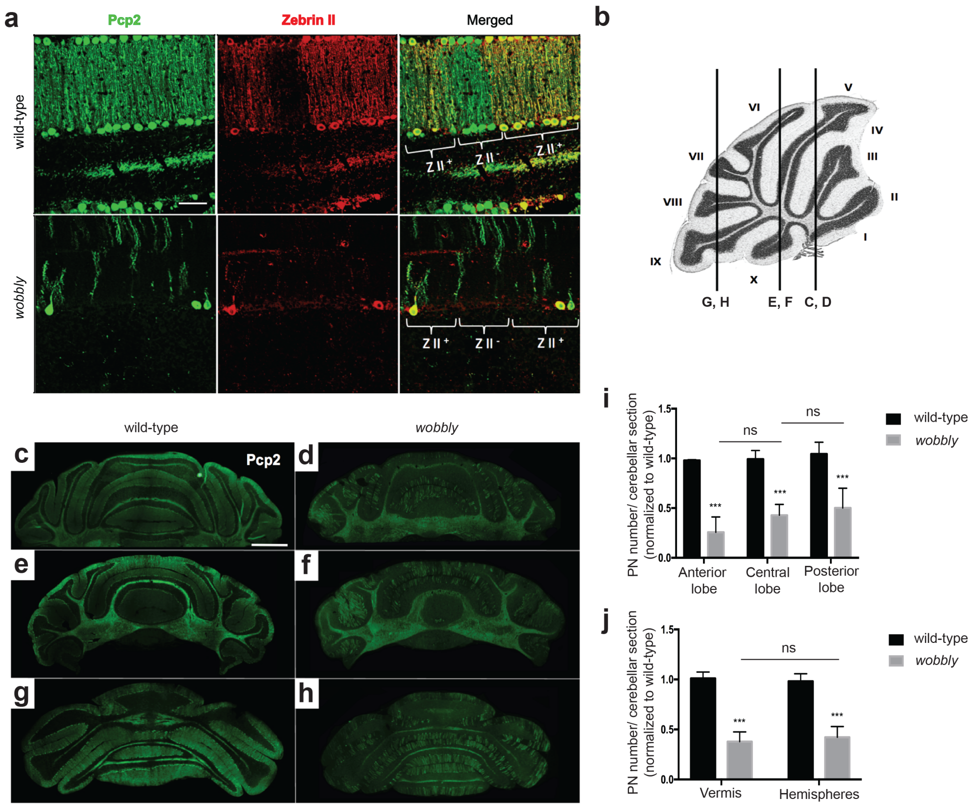

Supplementary Figure 4. Patterned and uniform PN degeneration in *wobbly* mice. (**a**) Immuno-labeling of coronal sections of P60 animals using Pcp2 and Zebrin II antibodies reveals a striped pattern of Zebrin II expression (ZII^{+} and ZII^{-} stripes). (**b**) Schematic representation of a sagittal cerebellar section where the vertical lines indicate the location of coronal sections in **c**-**h**. (**c**-**h**) Pcp2 immunofluorescence on coronal sections of P60 mice from the anterior (**c**, **d**), central (**e**, **f**) and posterior lobe (**g**, **h**). (**i**-**j**) PN quantification across cerebellar divisions (**i**) and vermis and hemispheres (**j**). Statistical evaluation: two-way ANOVA and Sidak's multiple comparisons test. Statistically significant differences are indicated (*** $P \leq 0.001$). Data represent mean ± SEM. Scale bars: **a**, 50 µm; **c**-**h**, 500 µm. $n = 5$ for each of the genotypes; representative images are shown.

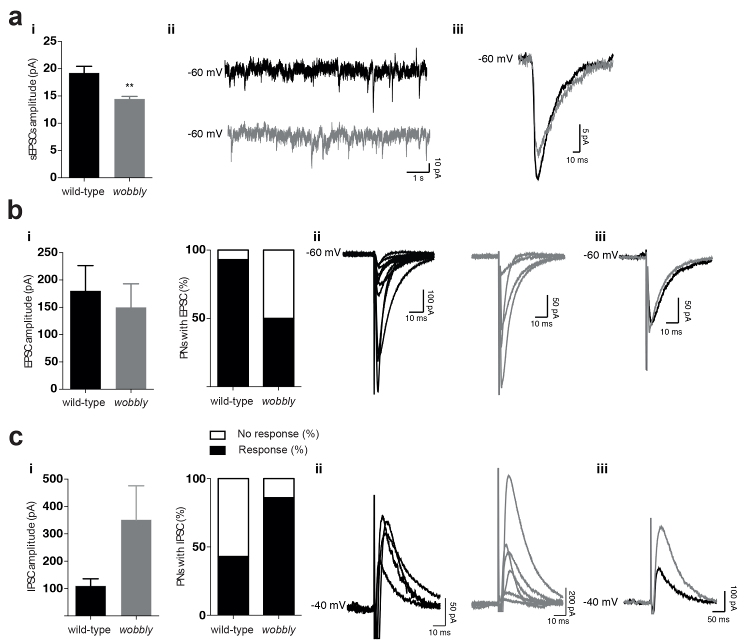

Supplementary Figure 5. Electrophysiological properties of *wobbly* PNs at P21-P24. (**a**) (**i**) sEPSC amplitude in wild-type PNs (n = 5) and *wobbly* PNs (n = 5). (**ii**) Example of sEPSCs traces from wild-type (black) and *wobbly* (grey) PNs recorded while holding PNs at -60 mV. (**iii**) Averaged sEPSCs recorded while holding *wobbly* (grey) and wild-type (black) PNs at -60 mV. (**b**) (**i**) Induced EPSC amplitude (left) and EPSC percentage (right) in wild-type (n = 14) and *wobbly* PNs (n = 4). (**ii**) Traces of each EPSC recorded while stimulating the parallel fibres while holding PNs at -60 mV. (**iii**) Averaged EPSCs induced by stimulating the parallel fibres while holding PNs at -60 mV. (**c**) (**i**) Induced IPSC amplitude (left) and IPSC percentage (right) in wild-type (n = 4) and *wobbly* PNs (n = 6). (**ii**) Traces of each IPSC recorded while stimulating the parallel fibres while holding PNs at -40 mV. (**iii**) Averaged IPSCs induced by stimulating the parallel fibres while holding PNs at -40 mV. Statistical evaluation: two-tailed t-test. Statistically significant differences are indicated (** $P \leq 0.01$). Data represent mean ± SEM.

a

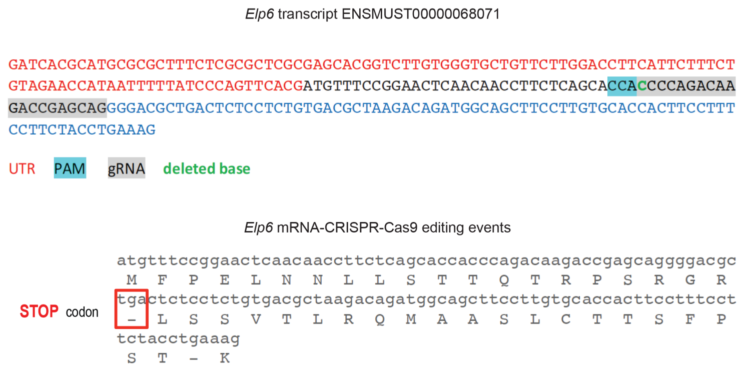

b

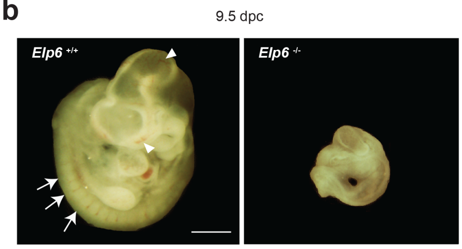

c

Age (dpc)	*Elp6* +/+	*Elp6* +/-	*Elp6* -/-
9.5	92	88	15
≥12.5	74	80	0
Newborn	68	72	0

Supplementary Figure 6. Early embryonic lethality of Elp6 loss. (**a**) *Elp6* targeting strategy via CRISPR-Cas9 system resulting in a nonsense mutation (introduction of a premature STOP codon). **b**) Gross morphology of a 9.5 dpc wild-type (*Elp6*$^{+/+}$) and Elp6 KO (*Elp6*$^{-/-}$) embryo (n = 15 for each of the genotypes; representative images are shown). Arrows point to somites and arrowheads indicate blood vessels in the wild-type embryo, with no corresponding structures found in the mutant embryo. Scale bar, 1 mm. (**c**) Offspring generated from Elp6 KO heterozygous crossings (*Elp6*$^{+/-}$). Numbers represent surviving embryos at three different stages of development. Abbreviations: UTR-untranslated region, PAM-photospacer adjacent motif, gRNA-guide RNA.

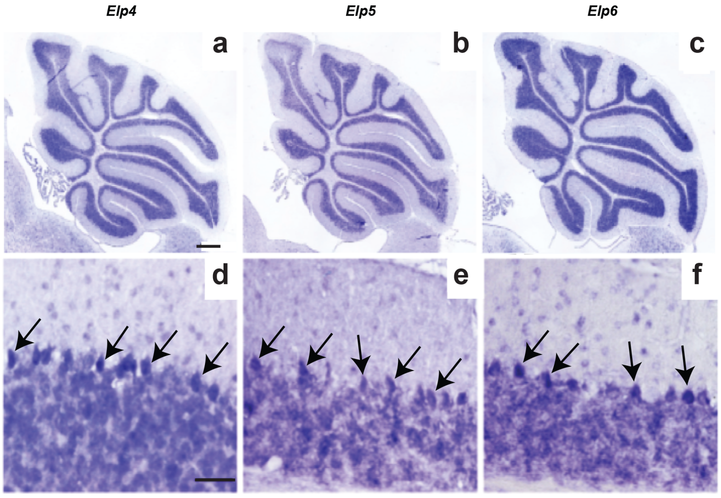

Supplementary Figure 7. Elp456 is widely expressed in the cerebellum. (**a**) *Elp4,* (**b**), *Elp5* and (**c**) *Elp6* RNA *in situ* hybridization on sagittal sections of P21 wild-type mouse cerebellum. (**d-f**) Magnification of the cerebellar cortex from (**a**) – (**c**) revealing the same expression pattern for all three probes. Arrows point to the expression of respective subunits in PNs. Scale bars: **a-c**, 500 µm, **d-f**, 50 µm. *n* = 5 for each of the RNA probes; representative images are shown.

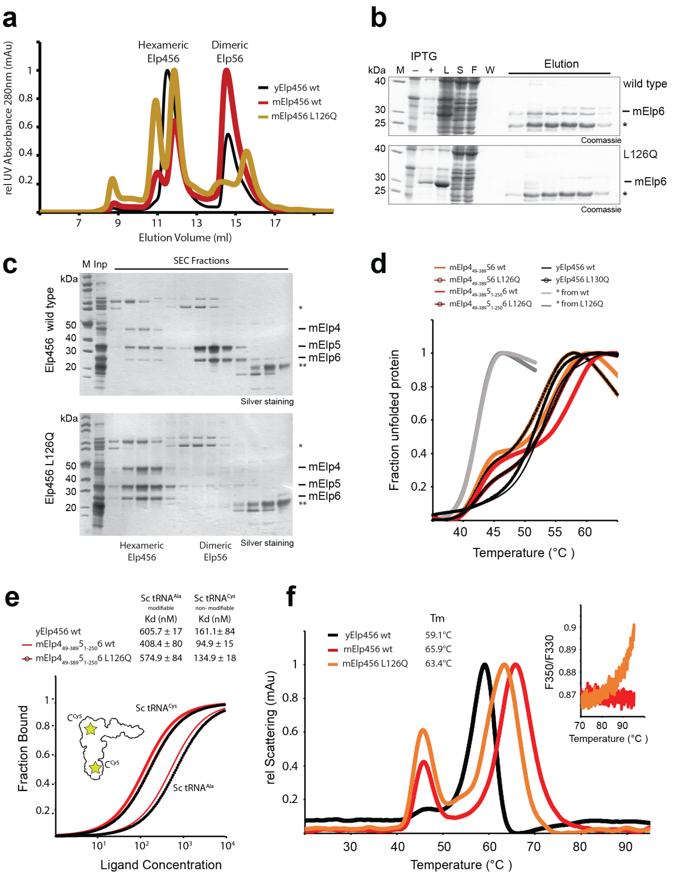

Supplementary Figure 8. Biochemical characterization of *mElp6L126Q*. (**a**) Size exclusion chromatography (SEC) of purified Elp456 complexes from yeast (black), mouse wild-type (red) and mouse Elp6L126Q (brown) expressed in bacteria. (**b**) SDS-PAGE analyses of His-tagged mElp6 wild-type (top) and L126Q mutant (bottom). Marker (M), lysate (L), supernatant (S), flow-through (F), wash (W) and eluted fractions are indicated. (**c**) SDS-PAGE analyses of triple His-tagged full length mElp456 carrying wild-type Elp6 (top) and Elp6L126Q (bottom). Individual proteins, sub-complexes and contaminations (*) are indicated. (**d**) Normalized Thermofluor curves from Fig. 4c. (**e**) Microscale thermophoresis (MST) analysis of purified Elp456 complexes from mouse wild-type (red) and mouse Elp6L126Q (black line) for alanine and cysteine tRNAs (tRNAAla tRNACys correspondingly). Calculated Kds and confidence values are indicated; $n = 3$. (**f**) Nano differential scanning fluorimetry (nanoDSF) analyses of purified Elp456 complexes from yeast (black), mouse wild-type (red) and mouse Elp6L126Q (brown). Scattering (first derivative) is plotted against temperature yeast (black), mouse wild-type (red) and mouse Elp6L126Q (brown). Inlet shows the F350/F330 ratio for murine wild-type and mutant mElp456.

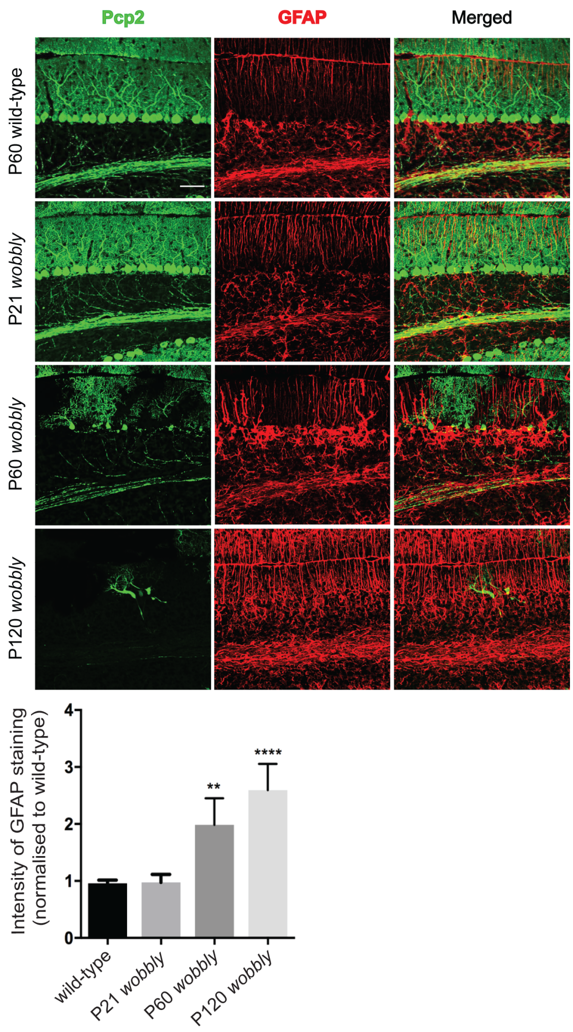

Supplementary Figure 9. Astrogliosis in *wobbly* mouse cerebella. Pcp2 and GFAP immuno-staining and GFAP quantification in *wobbly* and control animals. $n = 5$ for each of the genotypes; representative images are shown. Scale bar: 100 µm. Statistical evaluation: two-way ANOVA and Sidak's multiple comparisons test. Statistically significant differences are indicated (** $P \leq 0.01$; **** $P \leq 0.0001$). Data represent mean ± SEM.

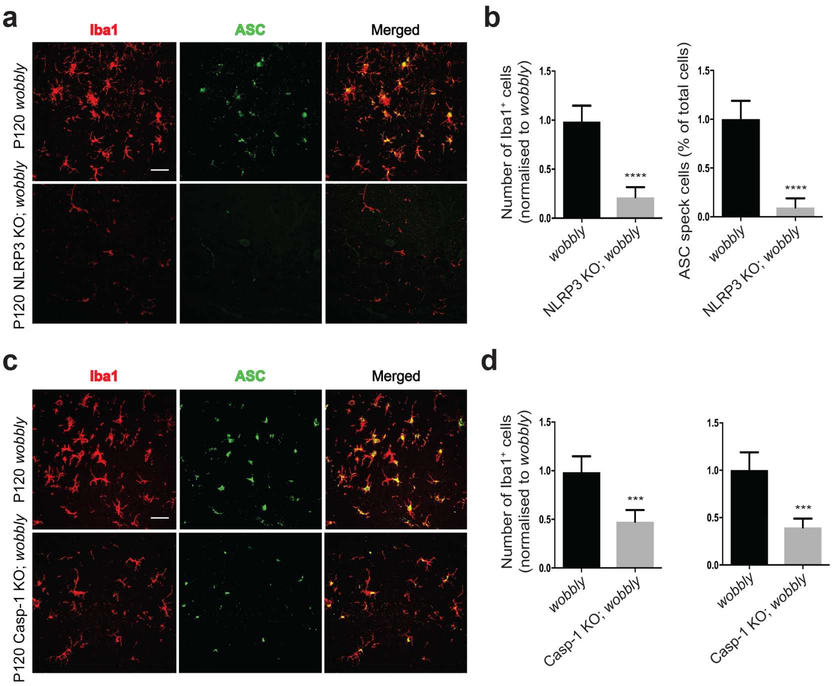

Supplementary Figure 10. Reduced inflammation in NLRP3 KO and caspase-1 KO *wobbly* mice. (**a**) Immunofluorescence of P120 NLRP3 KO; *wobbly* and control *wobbly* cerebella with Iba1 and ASC antibodies. (**b**) Quantification of microgliosis and ASC specks in (**a**). (**c**) Iba1 and ASC immunolabeling of caspase-1 (Casp-1) KO; *wobbly* and *wobbly* mice cerebellar sections. (**d**) Quantification of microgliosis and ASC specks in (**c**). $n = 5$ for each of the genotypes; representative images are shown. Statistical evaluation: two-tailed t-test. Scale bars: 10 µm. Statistically significant differences are indicated (*** $P \leq 0.001$; **** $P \leq 0.0001$). Data represent mean ± SEM.

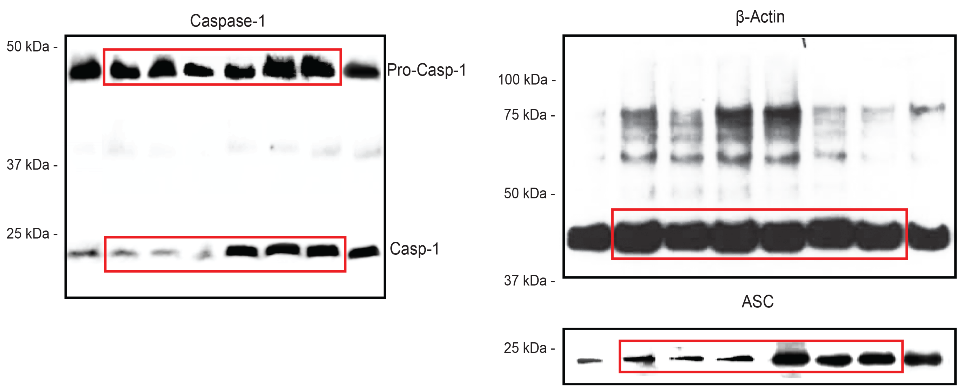

Supplementary Figure 11. Uncropped Western blots for Figure 6d.

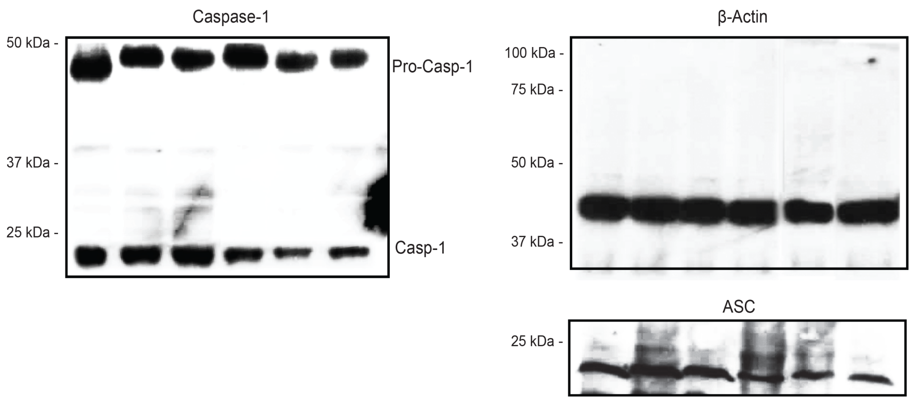

Supplementary Figure 12. Uncropped Western blots for Figure 7e.

Supplementary Table 1. Electrophysiological properties of PNs in *wobbly* mice

Passive and active membrane properties of *wobbly* and wild-type PNs at P21-24

	wild-type (n = 13) (mean ± SEM)	*wobbly* (n = 10) (mean ± SEM)	P value
Input resistance (MΩ)	107.6 ± 12	95.1 ± 20.1	$P = 0.2$
AP threshold (mV)	-44.5 ± 1.2	-40.1 ± 1.8	$P = 0.049$ *
AP onset (ms)	139.9 ± 40.8	87.8 ± 39.8	$P = 0.5$
AP peak (pA)	44.6 ± 1.7	44.5 ± 2.9	$P = 0.9$
AP half-width (ms)	0.3 ± 0.02	0.3 ± 0.02	$P = 0.5$
AP rise time (ms)	0.2 ± 0.02	0.2 ± 0.1	$P = 0.8$

sEPSCs properties of *wobbly* and wild-type PNs at P21-24

	wild-type (n = 5) (mean ± SEM)	*wobbly* (n = 5) (mean ± SEM)	P value
sEPSC frequency (Hz)	16.9 ± 2.4	15.2 ± 2.7	$P = 0.7$
sEPSC rise time (ms)	2.5 ± 0.2	2 ± 0.1	$P = 0.08$
sEPSC decay time (ms)	19.4 ± 2.8	15.2 ± 2.3	$P = 0.3$

Induced EPSC properties of *wobbly* and wild-type PNs at P21-24

	wild-type (n = 10) (mean ± SEM)	*wobbly* (n = 3) (mean ± SEM)	P value
EPSC rise time (ms)	1.2 ± 0.1	0.8 ± 0.2	$P = 0.1$
EPSC decay time (ms)	9.1 ± 1.4	6.4 ± 2.3	$P = 0.4$
PPR	1.6 ± 0.6	1.5 ± 0.1	$P = 0.3$

Induced IPSC properties of *wobbly* and wild-type PNs at P21-24

	wild-type (n = 5) (mean ± SEM)	*wobbly* (n = 6) (mean ± SEM)	P value
IPSC rise time (ms)	1.4 ± 0.3	1.6 ± 0.2	$P = 0.5$
IPSC decay time (ms)	23.2 ± 5.2	21.2 ± 3.6	$P = 0.7$

Statistical evaluation: two-tailed t-test. Statistically significant differences are indicated (* $P \leq 0.05$).

Supplementary Table 2. Dynamic mass spectrometer parameters for ribonucleosides

Nucleoside	Q1 (m/z)	Q3 (m/z)	Collision Energy (V)	Retention Time (min)
m^5C	258	126	15	5.5
s^2U	261	129	15	6.5
m^7G	298	166	18	8.8
ncm^5U	302	170	15	5.8
mcm^5U	317	185	15	8.2
